# Correlation between Nutrition and Symptoms: Nutritional Survey of Children with Autism Spectrum Disorder in Chongqing, China

**DOI:** 10.3390/nu8050294

**Published:** 2016-05-14

**Authors:** Xiao Liu, Juan Liu, Xueqin Xiong, Ting Yang, Nali Hou, Xiaohua Liang, Jie Chen, Qian Cheng, Tingyu Li

**Affiliations:** 1 Department of Child Health Care, Children’s Hospital of Chongqing Medical University, Chongqing 400014, China; caretoo@163.com (X.L.); liujuanyehai@163.com (J.L.); candyhnl@163.com (N.H.); 2 Children’s Nutrition Research Center, Hospital of Chongqing Medical University, Chongqing 400014, China; tingyang1206@126.com (T.Y.); liangxiaohua666@gmail.com (X.L.); jchen010@cqmu.edu.cn (J.C.); 3 Ministry of Education Key Laboratory of Child Development and Disorders, Key Laboratory of Pediatrics in Chongqing, China International Science and Technology Cooperation Base of Child Development and Critical Disorders, Chongqing 400014, China; 4 Pediatric Department of Clinical Medicine of Dazhou Vocational and Technical College, Dazhou 635001, China; m1868282131@163.com

**Keywords:** autism spectrum disorders, mealtime behavior, nutrient intake, biochemical assessment, vitamin A

## Abstract

Restricted diets and inadequate nutrient intake of children with autism spectrum disorder (ASD) have been reported. This study examined the nutritional statuses of children with ASD and the relationships between their behaviors and nutritional intake. A total of 154 children with ASD (age = 5.21 ± 1.83 years) and 73 typically-developing (TD) children (age = 4.83 ± 0.84 years) from Chongqing, China, were enrolled. The severity of ASD was evaluated using the Childhood Autism Rating Scale (CARS). The serum ferritin, folate, vitamin B12, 25(OH) vitamin D, and vitamin A concentrations in the children with ASD were determined. All participants underwent anthropometric examinations, dietary assessments, and questionnaire assessments about their feeding behaviors, and gastrointestinal symptoms. The Z_HA_, Z_WA_, and Z_BMIA_ were found to be significantly lower in the children with ASD compared with those without ASD. In addition, the percentages of children exhibiting severe picky eating and severe resistance to new foods, as well as those with a reported general impression of severe eating problems and constipation, were higher among the children with ASD. These children consumed significantly fewer macronutrients compared with the children without ASD. In addition, the children with ASD had the highest rate of vitamin A deficiency, followed by iron deficiency. After adjusting for sex, the vitamin A concentration was found to be negatively correlated with the CARS score (*r_s_* = −0.222, *p* = 0.021). No correlation between the ferritin, folate, vitamin D, or vitamin B12 concentration and the CARS score was found. These results suggest that reduced macronutrient intakes, severe feeding behavior issues, constipation, and vitamin A deficiency are quite common among children with ASD. Further, a low serum vitamin A level may be a risk factor for symptoms of ASD. However, the underlying mechanism should be further studied.

## 1. Introduction

Autism spectrum disorder (ASD) is characterized by deficits in social communication and social interactions, as well as restricted, repetitive patterns of behavior, interests, or activities. The nutritional management of children with ASD is a great challenge [1]. These children frequently have restricted diets limited to foods with specific tastes or textures or to specific types of foods [2,3,4,5]. Moreover, a high prevalence of gastrointestinal (GI) symptoms, such as persistent diarrhea and constipation, is common in children with ASD [6,7,8]. Feeding and defecation disturbances may affect their digestion and absorption functions, thereby affecting their nutritional statuses. However, studies investigating nutrient intake in children with ASD have produced conflicting results; the dietary intakes of macronutrients and some micronutrients in children with autism have been found to be lower, higher, or similar compared with the recommended amounts [9,10,11,12,13,14]. Meanwhile, a high rate of obesity has been observed among these children [15,16]. Considering that ASD is a highly heterogeneous disease, the discrepancies among these results might be due to differing gene-environment interaction mechanisms in children with ASD.

Notably, the relationship between ASD symptoms and nutrition has attracted research interest. For example, Meguid *et al.* have found that children with autism have significantly lower 25(OH) vitamin D (VD) and calcium levels, and have detected a significant positive correlation between the VD and calcium levels in these children [17]; however, the associated underlying mechanism has not yet been elucidated. Therefore, there is still a need to evaluate the nutritional statuses of the individuals in this specific population.

In the city of Chongqing, the youngest municipality in China, the economic conditions reflect the middle class Chinese population [18]. Few studies have reported the nutritional statuses of children with ASD in this area. Thus, the aims of the present study were as follows: to determine (1) whether the growth, mealtime behaviors, and GI symptoms of children with ASD differ from those of controls; (2) whether the levels of biochemical indices of nutrition are lower in children with ASD compared with the Chinese standards; and (3) whether a relationship exists between nutritional status (the vitamin A (VA), VD, vitamin B12 (VB12), ferritin (FER), hemoglobin (Hb), and folate (FOL) concentrations) and ASD symptoms. The results will help to guide the nutritional management of children with ASD.

## 2. Methods

### 2.1. Participants and Methods

#### 2.1.1. Inclusion/Exclusion Criteria

All children with ASD from six training institutions in Chongqing, which is located in the southwestern part of inland China, were recruited for this study. The diagnoses were made after a series of structured interviews were conducted by a psychologist and two developmental pediatricians at Children’s Hospital based on the criteria for autism defined in the Diagnostic and Statistical Manual of Mental Disorders [18], 5th Edition. The exclusion criteria were as follows: (1) diagnosis of Asperger syndrome, Rett syndrome, or another congenital disease, such as congenital heart disease, chronic seizures, or an acute or chronic affective disease during the previous three months; (2) any food or drug allergy; (3) use of any nutritional supplements during the previous six months; and (4) use of any medication to address behavior/focus/attention during the previous six months.

The typically-developing (TD) controls included 73 children sampled from one common kindergarten. This class was selected from among 38 kindergartens in the main urban district of Chongqing (at the lower intermediate socioeconomic level) to match the ASD families. The children were matched according to age, gender, the minority percentage, the family structure, and the parents’ education levels. The TD children had no abnormal histories of motor, language, or social developmental disorders, as determined according to the reports of the parents and teachers.

#### 2.1.2. Questionnaires

The caregivers of the children with ASD and the TD children followed the instructions provided by the trained investigator and completed the following questionnaires. 

The background questionnaire contained questions pertaining to demographic data (name, age, gender, nationality, and family structure), socioeconomic information (parents’ education levels and family annual income), and medical history.

The mealtime behavioral questionnaire included questions about oral function (hypersensitivity/hyposensitivity), eating problems (picking at food and refusal of new foods), chronic GI symptoms (abdominal pain, constipation, diarrhea, and vomiturition), and drug and food allergies. The caregivers were asked to report their impressions on the frequencies of mealtime behavioral issues by rating them according to the following scoring system: 0 = normal (never to seldom) or mild (sometimes); or 1 = severe (usually to always). Thus, the caregivers selected one of these two options to describe the severity of each problem.

The Childhood Autism Rating Scale (CARS) is commonly used as a diagnostic scale and for assessing ASD severity [19,20,21,22,23], and it was completed by the caregivers of the children with ASD. Total scores were calculated for each child with ASD.

#### 2.1.3. Anthropometric Measurements

Anthropometric examinations were conducted by three anthropometrists who were trained by and certified at the Department of Child Health Care of Children’s Hospital. Standardized procedures were conducted [24]. *Z*-scores were calculated for height-for-age (Z_WA_), weight-for-height (Z_HA_) and body mass index (BMI; Z_BMIA_) with WHO Anthro and AnthroPlus software (World Health Organization, 2009, Anthro for Personal Computers, Version 3.01: Software for Assessing Growth and Development of the World’s Children), using the WHO child growth standard 2005 version for children aged 0–5 years and the 2007 version for children and adolescents aged 5–19 years. *Z*-scores of less than −2 for Z_WA_, Z_HA_, and Z_BMIA_ were indicative of underweight, stunting/short stature, and wasting, respectively, while scores of higher than +2 were indicative of overweight, tall stature, and obesity.

#### 2.1.4. Physical Examinations for Malnutrition

Physical examinations were conducted by three pediatricians. All signs and symptoms, including nutrient deficiencies (such as Bitot’s spots, night blindness, and rickets), were recorded.

#### 2.1.5. Developmental Assessments

The Gesell Developmental Scale (GDS) has been revised and is widely used in China to assess neurodevelopment in children [25,26]. This scale was used to assess the cognition and behavioral development of the children in the present study, with the grouping of the items into the following five domains: adaptive behavior, gross motor, fine motor, language, and personal-social behavior. The standardized mean ± standard deviation (SD) of the developmental quotients (DQs) was 100 ± 15. Scores of lower than 70 were considered to indicate developmental delay. The tests were conducted by three trained developmental pediatricians.

#### 2.1.6. Dietary Intake Investigation

Dietary intake was evaluated using a 24-h food weighing method and two-day diet diaries, which were applied for one week. The participants ate in the dining hall of the institution or kindergarten for five weekdays, and they ate at home for two weekends. For the 24-h food weighing method, two trained anthropometrists spent an entire day in the kitchen of the institution weighing all types of food (uncooked and cooked), while six trained anthropometrists stayed with each participant to weigh all food that they consumed, including snacks. In addition, the caregivers were asked to complete each child’s two-day food diaries on the weekends; prior to this process, trained anthropometrists provided different emulational food models to the caregivers to demonstrate the description and estimation of food intake, and instructions were also provided for the standardized recording method.

Nutrient analysis software was used to analyze the dietary intake of the children (Huicheng Corporation, Shanghai, China). The macronutrient intakes per day are presented as the mean and SD. The recommended nutrient intakes (RNIs) and adequate intakes (AIs) for the different age groups according to the Chinese Nutrition Academy (2000) were used for these analyses [27]. All results were converted to percentages relative to the RNI or AI. “Inadequate intake of energy” was defined as <EAR. “Inadequate intake of protein and other nutrients” was defined as <RNI or AI.

#### 2.1.7. Levels of Biochemical Indices of Nutrition

Venous blood samples were collected from the participants before breakfast, and 2 mL of each sample was placed into an EDTA-coated tube, while 1 mL was placed into a separation gel coagulation tube and then immediately (within 2 h) transported to Children’s Hospital. A 50μL volume of whole blood was used to measure Hb using the hemiglobincyanide method (MAKER, China). The blood samples in the separation gel coagulation tubes were centrifuged at 3500× *g*/min for 3 min, and then sera were extracted for measurements of the C-reactive protein (CRP), FER, FOL, VB12, VD, and retinol concentrations.

The CRP concentration was measured by particle-enhanced immunoturbidimetry (Orion Corporation Orion Diagnostica, Espoo, Finland), and the FER, FOL, VB12, and VD concentrations were measured using chemiluminescence microparticle immunoassay (CMIA) kits (Abbott Ireland Diagnostics Division, Longford, Ireland). The retinol concentration was determined using high-performance liquid chromatography (HPLC) according to the method of Miller and Yang with slight modifications [28]. All biochemical indices were measured by examiners at the pediatric laboratory of Chongqing Medical University, China.

Anemia was defined as Hb < 110 g/L for children aged five months to six years and Hb < 115 g/L for children aged 6–11 years, according to the WHO criteria [29]. A CRP level of >8 mg/L was indicative of infection or inflammation according to the manufacturer’s recommendations (Roche Diagnostics). The manufacturer-defined deficiency levels for FER, FOL, VB12, and VD were lower than 21.8 ng/mL and for males and 4.63 ng/mL for females, 3.1 ng/mL, 187 pg/mL, and 9.5 ng/mL, respectively. In addition, a retinol concentration of <0.7 μmol/L was defined as vitamin A deficiency (VAD), according to the WHO criteria [30].

#### 2.1.8. Statistical Analysis

All analyses were conducted using SPSS Statistics software (version 17.0, SPSS Inc., Chicago, IL, USA). The Kolmogorov-Smirnov goodness-of-fit test was used to test the distribution of each data set for normality before analysis. Normally distributed data are presented as the mean ± SD, and non-normally distributed data are presented as the median (P25, P75). Further, the demographic, clinical, and nutritional characteristics are depicted as frequencies and percentages. Two-tailed Student’s *t*-test, the Chi-squared test and Fisher’s exact test were used to compare differences between the two groups. *P* values of <0.05 were considered statistically significant. Partial correlation analysis was performed to identify relationships between the nutrients and CARS scores.

#### 2.1.9. Ethical Clearance

The research was conducted according to the guidelines stated in the Declaration of Helsinki. All procedures were approved by the Institutional Review Board of Children’s Hospital (No. 058/201). Written informed consent was obtained from the parents prior to enrollment of the children.

## 3. Results

A total of 154 children with ASD (141 boys and 13 girls) and 73 TD children (67 boys and six girls) were recruited between August 2013 and October 2014. The children with ASD were 5.21 ± 1.83 years old, and the TD children were 4.83 ± 0.84 years old. A total of 96.1% and 95.1% of the children with ASD and the TD children, respectively, were of Han nationality. In addition, 80.5% and 87.7%, respectively, were from large families (with more than one child). Symptoms of Bitot’s spots, night blindness, and rickets were not observed in the physical examinations. All children underwent anthropometric examinations, growth assessments, questionnaire assessments, and dietary surveys. In addition, all children with ASD underwent biochemical testing for determination of their nutritional statuses, while none of the TD children underwent this invasive testing due to refusal of their caregivers. A total of 26.7% of the children with ASD were from poor families, with annual incomes of below 10,000 RMB, 27.9% were from families with annual incomes ranging from 10,000 to 40,000 RMB, and 45.5% were from families with annual incomes of above 40,000 RMB; these are the lower income levels in Chongqing. The demographic characteristics and GDS scores of the children are shown in Table 1.

The CARS score of the children with ASD was 34.2 ± 8.73. The percentages of these children exhibiting delays in adaptive behavior, gross motor, fine motor, language, and personal-social behavior were 91.6% (141/154), 81.2% (125/154), 75.3% (116/154), 98.1% (151/154), and 87.7% (135/154), respectively. These data indicated that most of the participants with ASD were developmentally delayed.

The growth assessment results are provided in Table 2. *Z*-scores for weight, height, and BMI, and the percentages of children exhibiting abnormal growth were calculated. All *Z*-scores for the children with ASD, including Z_HA_ (0.41 ± 1.10), Z_WA_ (−0.01 ± 1.12), and Z_BMIA_ (0.65 ± 1.17), were significantly lower than those for the TD children. Further, the percentage of children with stunting/short stature was significantly higher (7.14% *vs.* 0%) and the prevalence of obesity was significantly lower (9.74% *vs.* 20.54%) in the ASD group compared with the TD group. In addition, no significant between-group differences in the percentages of children with wasting, tall stature, or overweight were observed.

The mealtime behaviors and GI symptoms of the two groups were compared (Table 3). No significant differences were observed in the percentages of children with oral hyposensitivity or hypersensitivity, mild picky eating, mild resistance to new foods, vomiturition, or diarrhea between the ASD and TD groups. However, the percentages of those with severe picky eating, severe resistance to new foods, a reported general impression of severe eating problems, and constipation were significantly higher in the ASD group (26.0% *vs.* 11.0%, 9.0% *vs.* 1.4%, 32.5% *vs.* 13.7%, and 22.1% *vs.* 1.48%). In addition, a marginal difference in the reported general impression of mild eating problems was observed (*p* = 0.052466).

A comparison of macronutrient intake between the two groups is shown in Table 4. Lower average intakes of energy (87.44% *vs.* 119.89%), protein (87.39% *vs.* 115.67%), carbohydrates (84.09% *vs.* 101.24%) and fats (87.60% *vs.* 152.18%) were detected in the ASD group compared with the TD group. In addition, a markedly increased prevalence of inadequate macronutrient intake was detected in the ASD group. All of these differences were significant.

The daily intake of one important micronutrient, the vitamin A was calculated. The daily intakes of vitamin A in the children with and without ASD were 313.63 ± 220.66(142.47, 442.07) μg RE and 339.76 ± 151.39(211.98, 411.57) μg RE, respectively, which were 52.88% and 57.08% of the RNI. The difference between the two groups was not significant (*U* = −1.315, *p* = 0.188).

Table 5 presents the levels of biochemical indices in the children with ASD. The CRP level in these children was <8 mg/L. The greatest deficiency was observed for VA (77.9%), followed by iron (20%). There were no significant deficiencies in VB12 (4.8%), VD (3.4%), or FOL (2.1%).

Table 6 lists the comparisons of vitamin A levels and vitamin A deficiency rates in the children with and without ASD in different age groups. The vitamin A levels of children younger than six years old were significantly lower than those of TD.

The partial correlations between the CARS scores and levels of biochemical indices of nutrition are listed in Table 7. After adjusting for sex, significant gender differences were detected in the ASD group. Only the correlation between the VA concentration and CARS score was significant (*r**_s_*** = −0.222, *p* = 0.021).

## 4. Discussion

In this cross-sectional study, we prospectively assessed the nutritional statuses, mealtime behaviors, and GI symptoms of children with ASD and the relationships between the VA, VD, VB12, FER, Hb, and FOL concentrations and ASD symptoms. The results indicated that increased percentages of the children with ASD had abnormal growth, mealtime behavioral issues, and constipation, as well as a reduced daily nutritional intake and lower VA concentration. The VA concentration was found to be negatively correlated with the CARS score in this group of children. The results of this study confirm our hypothesis.

### 4.1. Worse Growth Assessments and Lower Daily Nutritional Intake in ASD

The children with ASD in our study had significantly lower Z_WA_, Z_HA_, and Z_BMIA_ values, as well as a lower rate of obesity and a higher rate of stunting/short stature, compared with those without ASD. As weight is a sensitive nutritional index that is related to height, the levels of the biochemical indices in the children with ASD indicated that they had acute or chronic malnutrition. Similarly, Mari-Bauset *et al.* reported that autistic children in Spain had lower BMIs than the study controls [31]. In addition, Hyman has suggested that children with ASD aged 5–11 years have a higher incidence of being underweight, whereas those aged 2–5 years have higher incidences of overweight and obesity compared with those without ASD [32]. However, Sun *et al.* found that children with ASD in Harbin, China, had higher mean BMI, Z_WH_, and Z_BMIA_ and suggested that among autistic children, there was a possibility of becoming overweight and an increased risk of being obese [33]. Further, de Vinck-Baroody determined that the prevalence of obesity in children with ASD was higher than that in a national sample [34]. In addition, Dreyer found that children with ASD aged 10–17 years in the U.S. were more likely to be obese than those without ASD [35].

In general, the different trends in growth deviation are related to their daily nutritional intakes, activities, and genetic backgrounds. In the present study, the growth deviation can be interpreted based on the daily nutritional intakes. We found that the daily intakes of macronutrients (energy, proteins, fats, and carbohydrates) were lower in the children with ASD relative to the RNIs and the daily intakes of the TD children (Table 4). These results might be partially related to the restricted mealtime behaviors and lower socioeconomic backgrounds of the children with ASD. Our participants were mainly from poor families with annual incomes of below 40,000 RMB, which is quite low compared to the abovementioned surveys conducted in northeast China and the West. The cost of education for the children with ASD often decreased the quality of the meals served at home, potentially resulting in abnormal growth. Additionally, we noticed discrepancies among the nutritional intakes reported in the literature. Sun *et al.* observed no notable differences in the mean protein, carbohydrate or fat intakes of children with and without ASD in a survey conducted in northeast China [34]. However, in reviewing the results of this study, it was difficult to explain the higher BMI, Z_WH_ and Z_BMIA_ observed in the children with ASD. Hyman reported that children with ASD and matched controls obtained similar amounts of nutrients from their foods; however, the children with ASD (aged 4–8 years) consumed significantly less energy, and the high proportion of obesity observed in those who were in preschool was thought to be related to eating snacks and being less active during therapeutic activities [33].

Since the ASD group in this study demonstrated inadequate macronutrient intake, dietary intake should be increased at the training centers in Chongqing.

### 4.2. Mealtime Behaviors in Children with ASD

Martins reported that children with autism were only marginally more likely to exhibit picky eating behaviors and that the percentage of these children exhibiting ritualistic feeding behaviors was equivalent to those of their siblings or matched TD children in Australia [36]. In contrast, the proportions of children with ASD with severe picky eating, severe resistance to new foods, and a reported general impression of severe eating problems were significantly increased in our study. However, no noticeable differences were detected in the percentages of children exhibiting general mealtime behavioral issues (oral sensitivity, a reported general impression of mild eating problems, mild picky eating, or mild resistance to new foods) between the children with and without ASD, indicating that the general mealtime behaviors were similar among the children in the various age groups. However, the proportion of children for whom a general impression of mild eating problems was reported exhibited a marginally significant difference between the two groups. Thus, a larger sample size might have resulted in the identification of a greater proportion of children with ASD exhibiting mild mealtime behavioral issues. Similar to our results, many studies have reported significant differences in feeding and eating behaviors between children with and without ASD. In the U.S., more children with ASD have been found to be picky eaters, to be resistant to new foods, and to consume limited foods compared with controls [37,38,39,40,41]. Furthermore, parents of children with ASD have reported significantly increased levels of parental stress and more behavioral problems in their children [42]. Therefore, mealtime behavioral management is basic and important in clinical practice. The food preferences of children with ASD have been attributed to the influences of sensory factors, such as sensory sensitivity [38], and family food preferences [43]. However, elucidation of the true underlying mechanism requires further study.

### 4.3. GI Symptoms in ASD

Our results indicated that among all of the GI symptoms observed, the rate of constipation was significantly different between the two groups of children, with a higher incidence in the children with ASD. Considering the poor language skills of these children, these results were based on stool samples that could be easily observed by the parents. The percentage of children determined to have constipation was indeed credible; however, the rates of other GI symptoms, such as nausea and abdominal pain, may have been underestimated. Similar results have been reported by several research groups. For example, Levy SE found significant abnormalities in stool consistency among children with ASD compared with those without ASD [44], and Wang reported that the two most common problems experienced by children with ASD were constipation and chronic diarrhea [45]. Further, Chaidez V indicated that children with ASD who had frequent GI symptoms scored worse for core symptoms compared with those without frequent GI symptoms [46]. GI symptoms are common in the children with ASD. A population-based study has suggested that a neurobehavioral rather than a primary organic GI etiology may account for the higher incidence of these symptoms in children with autism [7].

### 4.4. Relationship between Micronutrient Levels and CARS Score

Anemia was one of the top nutritional diseases observed among the children, and iron deficiency was the main underlying cause [47,48]. Notably, iron deficiency and iron deficiency anemia have been reported to be more common in individuals with developmental and psychiatric disorders [49,50]. The percentage of children with ASD who had anemia (2.68%) in this study is lower than the average percentage (23.5%) previously reported in the Chongqing suburb [51]. FER deficiency, which is an indirect index of iron deficiency, was more common than VB12 (4.8%) and FOL (2.1%) deficiencies. Although negative relationships between these deficiencies and the CARS score were detected, no significant differences were found.

Similarly, in our study, a negative relationship between VD deficiency and the CARS score was observed, but no significant differences were identified between the two groups of children. The relationship between VD deficiency and ASD has received increased attention in recent studies [52,53]. Cannell has explained that experiments provide compelling data on the roles of VD in brain proliferation, differentiation, neurotropism, neuroprotection, neurotransmission, and neuroplasticity [54]. Adequate, and perhaps pharmacological, doses of VD may have therapeutic effects on the core symptoms of autism [55]. Although the underlying mechanism is unclear, VD is believed to play an important role in ASD.

In our investigation, VAD was the most prevalent nutrient deficiency observed among the children with ASD. Compared with the VAD rate of 8.9% among Chongqing preschoolers, as previously determined by our team [56], the VAD rate among the children with ASD in the present study was higher. Even compared with VAD rate in TD at lower intermediate socioeconomic status in our study, VAD in children with ASD younger than 6 years old were significantly more popular. Considering the age, gender, the minority percentage, the family structure, and the parents’ education levels in different age groups had been paired in children with and without ASD, the higher rates of VAD were distinguished in ASD in deed.

The reason of VAD should be considered seriously. Often it may be interpreted by inadequate intake. It is known that the vitamin A level in people at lower socioeconomic status are generally lower than those at average and higher economic level, and we found the intakes of vitamin A of children with ASD was at the same level as that of TD with lower intermediate socioeconomic status. So, it could be inferred that their vitamin A levels were lower than those at the average economic level. However, on the other hand, it was hard to explain the significant difference in rates of VAD between children with ASD and TD in most age groups in our study. Since there were no differences in intakes between them, and from another point of view, it confirmed that the mealtime behaviors had no more influence on the intakes of the both groups in our study.

A related analysis revealed that the VA concentration was negatively correlated with the CARS score, indicating that a lower VA concentration was associated with increased ASD severity. However, we did not identify the specific causes of this association. Whether the more serious the disease (the higher score of CARS) causes or aggravates VAD, or the VAD causes or aggravates the symptoms of ASD? We have no more evidence to confirm it. Whether the universal and significant VAD in children with ASD can aggravate the symptoms of ASD, research seldom gave strong evidence. Visual loss [57] and xerophthalmia [58], which are typical symptoms of VAD, have been reported in autistic children who consume a large amount of foods lacking VA due to specific food restrictions. A higher prevalence of VAD due to the reduced daily intakes and biochemical levels in children with ASD has also been identified in another Chinese sample [33]. However, to our knowledge, few studies have investigated the relationship between the VA concentration and the core ASD symptoms. Megson has indicated that VA supplementation may be effective for treating the symptoms of autism; this author concluded that a specific gene is absent in patients with ASD [59]. However, this hypothesis has not yet been confirmed.

VA is a compound belonging to the retinoid family, which includes natural and synthetic compounds. Retinoids, as VA derivatives, are known to be involved in a complex signaling pathway. Animal studies of the developmental effects of retinoid signaling on the embryonic or early post-natal brain, as well as the effects of disruption of the retinoid signaling pathways, have demonstrated their involvement in the regulation of synaptic plasticity and associated learning and memory behaviors [60]. Our previous study has revealed that marginal VAD beginning during the embryonic period results in impaired learning, memory and long-term potentiation in young rats [61]. Other previous studies have confirmed the presence of positive correlations of the cord serum VA level with motor DQ and growth rate among preschool children [62], and of the VA level in pregnant women with the cognitive functioning of their children at two years of age [63]. The brain impairment might not be reversible if VA supplementation is initiated after the critical period for hippocampus development had already occurred. Thus, VAD during post-natal development may damage cognitive functioning. Therefore, we hypothesize that VA plays a role in the mechanism of ASD development. Whether and how the retinoid pathway is altered in children with ASD and in cell cultures *in vitro* will be assessed in future studies.

### 4.5. Limitalions of This Study

Our study had some limitations. First, and foremost, no biochemical results were obtained for the TD children. Although we attempted to perform biochemical testing on these children, we ultimately did not due to refusal of the caregivers, resulting in a lack of direct reference values for nutrient deficiency in the TD children. Therefore, we used indirect references from the database of Children’s Hospital, Chongqing Medical University. However, the indirect references represent average (from the minimum to the maximum) levels, whereas the TD children came from families at lower-to-intermediate socioeconomic levels. Whether, and how, the socioeconomic level affects the biochemical results remain uncertain; thus, it is not known whether there were differences between the direct and indirect references that could have reduced the reliability of our results. Second, the biochemical indices were not analyzed in the dietary survey because of limitations of the software. If they had been compared, the results would have been more complete. Third, the sample size was relatively small. Therefore, the present results should be confirmed in a larger-scale prospective study. Finally, ASD was only clinically diagnosed. Standardized diagnostic scales, such as the Autism Diagnostic Observation Schedule (ADOS) and the Autism Diagnostic Interview-revised (ADI-R), were not officially used in China during the study period.

## 5. Conclusions

The higher rate of malnutrition among the children with ASD from Chongqing, China, may have resulted from lower daily intakes of macronutrients (energy, proteins, fats, and carbohydrates). Therefore, corresponding nutrient management should be implemented. Furthermore, higher rates of severe mealtime behavioral issues, constipation and VAD were confirmed among the children with ASD. The VA level was found to be negatively associated with the severity of ASD, indicating that a low serum VA level may be a risk factor for behavioral symptoms in these children. Future research should be conducted to investigate the metabolic mechanism underlying VAD in ASD.

## Figures and Tables

**Table 1:** Demographic characteristics of children with ASD and TD.

Characteristic	ASD	Control (TD)
Age (years), mean ± SD (P25, P75)	5.21 ± 1.83 (3.85, 6.33)	4.83 ± 0.84 (4.41, 5.75)
Male, % ^a^	91.6 (141/154)	91.8 (67/73)
Age group (years), % (*n*/N) ^a^		
<3, % (*n*/N) ^a^	92.9 (13/14)	100 (5/5)
3–, % (*n*/N) ^a^	87.1 (27/31)	85.7 (18/21)
4–, % (*n*/N) ^a^	93.3 (28/30)	95.0 (19/20)
5–, % (*n*/N) ^a^	97.0 (32/33)	93.8 (15/16)
6–, % (*n*/N) ^a^	84.0 (21/25)	81.8 (9/11)
7–9, % (*n*/N)	95.2 (20/21)	0
Minority (not Han), % ^a^	3.9 (6/154)	4.1 (3/73)
Father’s educational levels, % ^a^		
Illiterate/elementary/middle school	29.9 (46/154)	23.3 (17/73)
High school	24.7 (38/154)	22.0 (16/73)
College or above	45.5 (70/154)	54.8 (40/73)
Mother’s educational levels, % ^a^		
Illiterate/elementary/middle school	33.8 (52/154)	31.5 (23/73)
High school	23.4 (36/154)	20.5 (15/73)
College or above	42.9 (66/154)	47.9 (35/73)
Nuclear family, % ^a^	19.5 (30/154)	12.3 (9/73)
Total scores of CARS, mean ± SD (P25, P75)	34.2 ± 8.73 (27.5, 39.0)	-
GDS scores		
adaptive behavior, mean ± SD (P25, P75)	43.8 ± 17.2 (31.5, 56.5)	-
gross motor, mean ± SD (P25, P75)	54.8 ± 19.1 (41.0, 66.0)	-
fine motor, mean ± SD (P25, P75)	52.4 ± 19.2 (38.5, 69.5)	-
language, mean ± SD (P25, P75)	32.8 ± 15.5 (21.5, 42.0)	-
personal-social ability, mean ± SD (P25, P75)	46.0 ± 17.9 (33, 57)	-

^a^ No significant difference was detected using the *X*^2^ test or Fisher’s exact test/*t*-test.

**Table 2:** Comparison of growth assessment results between children with ASD and TD.

Growth Assessment	ASD	TD
Z_HA_ *, mean ± SD	0.41 ± 1.10	1.06 ± 1.02
Z_WA_ *, mean ± SD	−0.01 ± 1.12	0.58 ± 1.09
Z_BMIA_ *, mean ± SD	0.65 ± 1.17	1.39 ± 1.00
stunting/short stature(Z_HA_ < −2), % *	7.14 (11/154)	0 (0/73)
underweight(Z_WA_ < −2), % ^a^	2.60 (5/154)	0 (0/73)
wasting(Z_BMIA_ < −2), % ^a^	0.01 (2/154)	0 (0/73)
tall stature (Z_HA_ > 2), % ^a^	2.60 (4/154)	5.48 (4/73)
overweight (Z_WA_ > 2), % ^a^	7.79 (12/154)	13.69 (10/73)
obesity (Z_BMIA_ > 2), % *	9.74 (15/154)	20.54 (15/73)

^a^ No significant difference was detected using the *X*^2^ test or Fisher’s exact test/*t-*test; * *p* < 0.05 using the *X*^2^ test or Fisher’s exact test/*t*-test.

**Table 3:** Comparison of mealtime behaviors between children with ASD and TD.

Mealtime Behavior	ASD	TD	*p*
Oral hyposensitivity, %	20.8 (32/154)	21.9 (16/73)	0.844434
Oral hypersensitivity, %	29.2 (45/154)	21.9 (16/73)	0.246313
Mild picky eater, %	41.6 (64/154)	32.9 (24/73)	0.209848
Severe picky eater, %	26.0 (40/154)	11.0 (8/73)	0.009661 *
Mild resistance of new food, %	14.5 (37/154)	20.5 (15/73)	0.560274
Severe resistances of new food, %	9.0 (14/154)	1.4 (1/73)	0.028725*
Total impression of mild eating problems, %	37.7 (58/154)	24.7 (18/73)	0.052466
Total impression of severe eating Problems, %	32.5 (50/154)	13.7 (10/73)	0.002742 *
Vomiturition, %	2.6 (4/154)	2.7 (2/73)	0.950215
Diarrhea, %	2.6 (4/154)	0	0.164746
Constipation, %	22.1 (34/154)	1.4 (1/73)	0.000055 *

* *p* < 0.05 using the *X*^2^ test.

**Table 4:** Comparison of macronutrient intake between children with ASD and TD.

Nutrients	ASD		NT		*p* (Comparison of Mean ± SD)	*p* (Comparison of %)
	Mean ± SD	% of RNI or AI	Mean ± SD	% of RNI or AI		
Energy, kcal *	1376.76 ± 484.93	87.44%	1869.23 ± 529.43	119.89%	0.0001	0.0005
Protein, g *	47.14 ± 17.02	87.39%	61.54 ± 18.82	115.67%	0.0014	<0.0001
Fat, g *	45.97 ± 28.98	87.60%	79.27 ± 30.30	152.18%	<0.0001	<0.0001
CHO, g *	198.45 ± 72.90	84.09%	235.25 ± 61.93	101.24%	0.0284	0.0269

* *p* < 0.05.

**Table 5:** Levels of biochemical indices of nutrition in children with ASD.

Nutrients	Age	Mean ± SD	% of Deficiency
Ferritin(ng/mL)	<18 years	40.16 ± 25.14	20
Folate (ng/mL)	<18 years	9.07 ± 3.84	2.1
Vitamin B12 (pg/mL)	<18 years	641.28 ± 349.78	4.8
25-OH Vitamin D (ng/mL)	<18 years	22.55 ± 7.43	3.4
Vitamin A(μmol/L)	<18 years	0.61 ± 0.21	77.9
Hemoglobin (g/L)	<6 years	130.92 ± 9.43	2.08
	≥6 years	135.93 ± 8.09	0

**Table 6:** Vitamin A levels and vitamin A deficiency rates in children with ASD and TD.

Age Group (Years)	ASD		TD	
	VA (Mean ± SD) (μmol/L)	VAD% (*n*/N)	VA (Mean ± SD) (μmol/L)	VAD% (*n*/N)
<3	0.5992 ± 0.0467	71.4(10/14)	0.5245 ± 0.0233 *	60.0(3/5)
3–	0.6448 ± 0.0434	71.0(22/31)	0.8685 ± 0.0825 *	52.4(11/21)
4–	0.5551 ± 0.0372	80.0(24/30)	0.6553 ± 0.0281 *	65.0(13/20)
5–	0.5530 ± 0.0361	72.7(24/33)	0.6809 ± 0.0354 *	56.3(9/16)
6–	0.5860 ± 0.0386	84.0(21/25)	0.5869 ± 0.0387	81.8(9/11)
7–	0.6623 ± 0.0558	57.1(12/21)	-	-

* *p* < 0.05 using *t*-test in the same age groups.

**Table 7:** Partial correlation analysis between CARS scores and levels of biochemical indices of nutrition.

Nutrients	*r_s_*	*p*
Vitamin A	−0.222	0.021*
Ferritin	−0.017	0.910
Folate	−0.252	0.095
Vitamin B12	−0.236	0.118
Vitamin D	−0.022	0.883

* *p* < 0.05.
